# The skills that help employees adapt: Empirical validation of a four-category framework

**DOI:** 10.1371/journal.pone.0282074

**Published:** 2023-02-24

**Authors:** Oscar Ybarra

**Affiliations:** Gies College of Business, Champaign, Illinois, United States of America; Jacobs University Bremen, GERMANY

## Abstract

Globalization, technological advances, economic and geopolitical shocks, pandemics, and any number of novel or unanticipated events have one thing in common: they represent change and require dynamic responses and adaptation from organizations, teams, and individuals. A critical resource for individuals to be adaptive are broad skills relevant to varied organizational conditions. These adaptive skills have been discussed in diverse venues but rarely in the organizational literature. Also, most, if not all, of extant conceptual frameworks related to adaptive skills remain unvalidated. The purpose of this research was to organize these skills, define and situate them in the relevant organizational and psychological literatures, and empirically test a proposed four-category framework. The experimental results supported the C+MAC framework, as skills were better categorized in terms of their theoretically related category. Additionally, the four-category framework proved a better fit to the skills compared to an influential, alternative model. The findings’ implications are discussed, noting how an empirically validated framework can facilitate understanding of how individuals engage with organizational environments and organizations get their work done.

## Introduction

Turbulent times for organizations are not new and will likely continue if not accelerate in the future [1–4]. From the great recession of just a few years ago, to the current pandemic and post-pandemic reality, a looming climate crisis destined to affect many industries, continued pressures from unanticipated global events and competition, and rapid technological improvements, many organizations will continue to face an operating environment that is turbulent and competitive. These factors create uncertainty and doubt about organizations’ existence [5], necessitating flexible structures and processes [6], as well as learning and adapting [e.g., 7]. To be nimble and adaptive, though, organizations now more than ever need their building blocks—the employees who are at the heart of the transformation process—to be adaptive.

### Adaptation in the organizational literature

Before discussing individual adaptation, it is important to briefly review adaptation in the broader organizational literature. This review is not comprehensive but intended to highlight the significance of the adaptation concept as well as its wide application.

#### Organizational adaptation

At the macro level, the assumption that organizations are adaptive is apparent in the foundations of organization theory [8], open systems theory [9], in behavioral [10] and resource views of the firm [11], and in theories of organizational learning [12] (for a recent review see [13]). Some organizational scholars have even suggested that adaptation is the central concern of strategic management [14]. Normative approaches to organization design have also been proposed, using empirical and modeling techniques that are aimed at achieving congruence of mission and strategy with an organization’s operating environment [e.g., 15].

#### Group / team adaptation

Much of the work in organizations gets done by teams. At this mezzo level of analysis, the concept of adaptation has seen rapid expansion in the last twenty or so years. Group or team adaptation has been broadly discussed from an evolutionary perspective, where the fit between member expertise and the challenges confronting the group is seen as a basis for adaptive leadership [16]. But most attention has been paid by organizational and human factors scholars, who have developed several theoretical models to describe team adaptation [2, 3, 17–19]. For example, Burke and colleagues [2] conceive of team adaptation broadly as an ongoing emergent phenomenon that results from dynamic and recursive, individual and dyadic team member actions aimed at detecting and responding to change to produce functional outcomes. Maynard, Kennedy, and Sommer [3] see team adaptation as benefitting from a requisite team capacity (member characteristics, member adaptability but also team structures and task features) and effective team process (action, transition, interpersonal; see [20]). Christian et al. [17] propose that team process and other emergent states will vary as a function of the event (adaptive stimulus) the team is adapting to, which can vary in origin (internal or external to the organization) and duration (temporary or sustained). More recent work on team adaptation has begun to empirically test various aspects of the process, such as transactive memory systems and implicit coordination [21], the different phases of team adaptation [22], shared team mental models [e.g., 23], in-action team reflection [24], and how team leadership interacts with team behavioral interaction patterns [25].

#### Individual adaptation

The deployment of teams is a way for organizations to adapt to dynamic environments [2, 3, 26]. Individuals of course make up teams, and models of team adaptation do consider individuals as inputs to team adaptation [e.g., 2, 3, 27]). But the adaptation of individuals should be viewed as an independent process that can interact with other aspects of the organizational transformation process (tasks & goals, other formal structures, the informal organizational structure), resources, technology, and other social relational patterns [cf. 28].

A variety of research at the individual level is relevant to adaptation. For example, it is proposed that leaders are adaptive to the extent they can deploy behaviors suited to changing organizational contexts [29], a stance in line with older contingency theories of leadership (for a review see [30]). Many studies also exist on how aspects of the person fit with the organization broadly construed, for example, in terms of the employee and the organization, employee and the job, and employee and the group (for a review see [31]). Other individual approaches have focused on the psychological reactions individuals have and the decisions they make to try to lessen the effects of negative work environments [32]. This work is related to coping [33, 34], which is seen as adaptive to the extent it is proactive and problem-focused. In their I-ADAPT theory, Ployhart and Bliese [35] additionally discuss the areas of strategy selection, reactions to organizational change, and task performance as ways in which individuals attempt to adapt.

In terms of task performance, research over the last twenty years has begun to frame it as adaptive performance, generally acknowledging that the task environment for individuals is not static but brings with it different changes and disruptions they must adjust to [36]. Adaptive performance has to do with behaviors a person can engage in to respond to, or in anticipation of, changes related to their work [37], yet is considered distinct from the performance of job-related tasks and the meeting of contextual or other organizational expectations [36]. Thus, adaptive performance is seen as a capacity to respond and adjust to different aspects of one’s work across different situations [27, 35], but also responding to changing requirements within the same task domain [e.g., 26]. Several studies have investigated predictors of adaptive performance, some of which include self-leadership [38], ambition [39], and several of the big 5 personality traits [40], for example.

This abbreviated review makes apparent the broad use of the adaptation concept in the organizational literature, whether the term is used explicitly or implicitly. If anything, a focus on adaptation seems to be accelerating. This makes sense given that adaptation is seen as important for individuals, teams, and whole organizations due to the varied factors (globalization, technological changes, economic shocks, crises) that continually create the need for organizations to respond to change in functional and effective ways.

### How skills fit into individual adaptation

Most employees have a portfolio of projects they are pursuing, each with its own set of task demands, constraints, social structures, and resources. Individual adaptation is thus not just about doing one task and responding to changes in task demands, but being engaged in several different tasks spread out across different projects that intersect with different collaborators, technologies, and time horizons.

The example of a typical faculty member serves as an illustration. A faculty member may have different classes to prepare and teach (each with its own material, teaching approach and students), be conducting research projects that use different methods and rely on different sources of funding and collaborators, be involved as a director of a center with its own set of projects, goals and donors, be on an editorial board, be part of different faculty or university committees, and even be attempting to advance a project she thinks will serve the overall college’s mission.

For many employees, the work context of adaptation can thus be quite diverse. And it is the demands and expectations for the whole portfolio that serve as the backdrop for recognizing a stimulus signaling the need for change. These change-related stimuli are likely to conform to one of several dimensions. For example, Pulakos et al. [41] sampled critical incidents from several jobs across many industry categories. Their analyses yielded eight dimensions related to the performance of tasks and jobs that require adaptation by individuals (also see [27]). These are: handling emergencies or crisis situations, handling work stress, creative problem solving, dealing with uncertain and unpredictable work situations, learning new work tasks, technologies, and procedures, the need to demonstrate interpersonal and cultural adaptability, and for certain jobs, demonstrating physically related adaptability. Many jobs, including the faculty member’s job, for example, will involve dealing with occasional crises, responding to work stress, adjusting to new technologies, and dealing with new people, or people whose preferences have changed or whose values differ. The “portfolio of work” proposal helps to further highlight that even if change is required for only one task or project at a time, this can have implications for the other elements of an individual’s work portfolio.

In the present framework, the basis for employee adaptation is the possession and use of a set of broad skills that are distinct from job-specific, technical skills. For example, the ability to work effectively in teams [3], or to communicate well [42], represent skills that apply across various organizational contexts. The distinction between narrow, technical skills and broad ones is analogous to life strategies in biology, in particular the strategies of specialists and generalists. The characteristics and behaviors of specialists are tuned to a small set of environmental conditions, but the characteristics and behaviors of generalists allow them to survive in a wider range of conditions as well as to respond more effectively to change [e.g., 43]. Employees of course are hired for certain technical skills to do their jobs, but the broader skills are critical.

The deployment of these broad skills, in different arrangements and weightings, allows individuals to start-up, add to, and keep their portfolio of projects operational. But these same skills are instrumental in allowing employees to respond to change in different dimensions to their work [41]. For example, the capacity to meet deadlines on a project will involve planning and clearly communicating expectations and standards of performance (to self or others), but if a change to the project is warranted because of unanticipated challenges, planning and clear communication will also matter as the employee has to describe the challenges they are facing, plan the needed changes, and justify any requested resources to get the project back on track. Thus, such broad skills play an important role in an individual’s general adaptive capacity. This view is consistent with frameworks that posit that KSAOs (knowledge, skills, abilities, & other individual characteristics) are what undergird an individual’s general ability to adapt [35, 41, 44], although what these skills are and why they matter for adaptation has not been elaborated (for a perspective that deals with more molar individual characteristics, see [45]).

Elaborating what these skills are is important because there is wide interest in them by companies [46], governments across the globe [47], education leaders [48], economists [49], and even the world economic forum [50]. But an examination of the organizational literature indicates little attention to and research on such skills (for an exception see [30]). Organizational research has examined some skills in isolation, such as emotional intelligence [e.g., 51, 52], leadership [42, 53], creativity [54], and cognition broadly defined [55], and there is some work that attempts to organize different competencies [56], but no comprehensive framework exists for understanding the variety of skills and how they relate to each other. Outside of the organizational literature, several frameworks for grouping these skills have been proposed, but this scholarly landscape is fragmented amid a cacophony of conceptual approaches. As important, no empirical validation has been provided for these frameworks.

The present research addresses these limitations and makes several contributions to the organizational and management literatures as well as the skills literature. First, the paper introduces to organizational scholars a comprehensive list of skills relevant to organizational behavior. It does this by connecting the skills to varied organizational and psychological research and phenomena. Second, it brings coherence to the wide-ranging skills literature, where little empirical validation has been offered for the proposed organizing schemes. Third, the proposed framework has several implications for different areas of organizational behavior. For example, in terms of leadership, adaptive leadership pays little attention to adaptation by subordinates. But the adaptability of all employees should lessen the need for hierarchical leadership [cf. 57]. A fourth contribution the paper makes is to help stich experience at the individual level to organizational behavior more generally. That is, although organizational performance and adaptation are influenced by top-down processes such as strategy and upper echelon cognition [58], the organization’s adaptation also depends on bottom-up processes emanating from adaptive employees. As Schein noted, for organizations to learn and adapt, they need employees who themselves are learning and adapting [59]. A final contribution the paper makes is to offer an empirically validated framework that can inform practice as well as scholarship. Organizational leaders use conceptual models to make sense of organizational events so they can predict and exert control. But as already noted, no scientifically based model exists that can help leaders and other organizational members understand the different skills and the role these skills play in a large swath of organizational behavior.

### Previous work on adaptive skills and a four-category framework (C+MAC) for moving forward

Here, these broader skills are referred to as *adaptive skills*, but they also go by names such as “21st century skills,” and they are discussed in more detail below. The critical questions for organizations, though, are: Do organizational leaders and other organizational members have agreed upon ways of defining and communicating about these skills to identify who has them and who is lacking them? Do they have ways of assessing and cultivating these skills? Affirmative answers to these questions depend, though, on having a valid model or conceptual framework for recognizing and understanding these skills.

A validated model of adaptive skills has been sought for some time now. Cisco, Intel, and Microsoft, for example, helped create a partnership to aid researchers in different countries try to develop frameworks to better understand adaptive (21st century) skills and to create interventions for cultivating them [60]. However, a look at the current state of the science suggests that despite a plethora of conceptual frameworks, limited progress has been made in defining and validating these skills. The present research thus proposes a conceptual framework for organizing, understanding, and testing these adaptive skills.

Any discussion of individuals adapting will likely invoke the concept of intelligence. Indeed, adaptation is considered a core aspect of what it means to be intelligent [61]. However, intelligence, as usually defined, assessed, and used in prediction is dominated by a focus on mental and cognitive processes, processes such as attention, memory, and reasoning. These processes matter for taking in information to understand organizational circumstances; the C in the C+MAC model to be discussed stands for cognition. But individuals also need other skills to be adaptive. They need motivation skills (M) [e.g., 62], action skills (A) [cf. 63, 64], and connection skills (C) [49].

#### Cognition

At its core, cognition is about gathering, organizing, and processing information to understand and draw insights about the circumstances one is in as well as to come up with solutions to challenges and unexpected events. The cognition skills are related to mental processes typically studied by cognitive psychologists and cognitive neuroscientists. These include, for example, attention, reasoning, judgment and decision-making, and executive functions that underlie planning and problem-solving, processes likely to load onto a fluid factor in intelligence research. But cognition skills also have to do with memory, knowledge, and expertise or crystallized processes, outcomes that reflect exposure to a society’s precepts as well as the received wisdom and knowledge of its cultural and educational institutions, and technical fields [65]. For individuals to be successful, they must not only be able to control their attention and to reason, but also leverage their memory and apply what they have learned [62]. Thus, for many jobs, some types of expertise are also required (e.g., in a field, in applications, processes, or systems), as well as some level of quantitative skill as data and numbers are used to understand and communicate about various aspects of an organization’s operations and performance. The skills that make up the cognition category in the C+MAC framework are: problem-solving, planning & reflection, expertise (in a discipline or field of study), analytical & detail focus, quantitative, and organizational ability. A point I will return to is that the skill *labels* can be adjusted to meet local usage; what matters more is that the skills be defined and related to an integrated, empirically supported conceptual framework.

#### Motivation

Motivation skills play a central role in adaptation as they represent the behaviors through which individuals create the energy needed to engage with work. Motivation skills allow individuals to persist through obstacles, bounce back from failures, and engage in work that needs to be done even when not of their choosing. It is through motivation that individuals keep learning and pursuing goals of value to themselves and to organizations.

Motivation has been referred to as “will do” whereas cognition as “can do” [62]. A person scoring high on the cognition skills may be able to process information well, but that does not mean they will do so in a relevant situation. Although intelligence research has kept motivational processes mostly separate from cognition [62], research points to their critical role in human performance. Findings indicate that motivation is critical for monitoring the learning one undertakes [66, 67], and that it plays a central role in the development of expertise [68]. Research that has examined intellectual/academic performance as well as measures of motivation as simultaneous predictors of performance indicates that the motivational factors are more important [69]. The skills that make up the motivation category in the C+MAC framework include intrinsic engagement, grit & work ethic, resilience, determination & purpose, dedication, and growth & mastery orientation.

#### Action

Adaptation cannot occur without action. Different individuals’ interpretations and conclusions about organizational events will adhere to reality to varying degrees. In addition, interpersonal influence in groups is not static but can change based on the competence individuals demonstrate [16]. So, the organizational “truths” that win out, such as what idea or approach to try for improving performance in one’s unit, are the ones whose viability have been tested and have garnered support. The process thus involves generating ideas but also taking action to execute and test the ideas, as well as leading efforts for change and influencing others through the results of one’s tests.

Although cognition matters for processing information to understand one’s surroundings, life is experienced as individuals act on their worlds. By planning things, a person can create expectations of what might be accomplished. But it is through taking action that things start to get done and the viability of ideas gets tested. Research has shown, for example, that a tendency to act is related to task motivation, which makes it more likely people attain their goals [70]. And research in the organizational literature has shown that more action and less cognitive analysis helps people problem-solve and create change in ill-defined, uncertain, and fast-paced environments [71, 72].

William James made the important observation that thinking is for doing [73]. But doing and being mindful of the consequences of one’s actions is critical for better thinking and for bringing about change [cf. 74]. Indeed, activity and manipulating the environment is posited to be a basic human need [75]. Further, work in cognitive science has posited that intelligent systems continually generate new information to improve their model of reality by relating an internal representation of the environment to changes in the environment brought about by their own behavior [63]. In the organizational psychology literature, the related notion of enactment of the environment [64] holds that through taking action a person starts linking together ideas and creating a model of the world that better captures the nature of the circumstances they are dealing with, but also what might not have been previously envisioned. Indeed, in his Opus Majus, Roger Bacon noted that without experience it is difficult to know anything well [76]. Thus, deploying action skills is critical for thinking effectively and attaining one’s goals. The skills that make up the action category in the C+MAC framework include leadership, influence, behavioral flexibility, initiative & bias for action, creative & entrepreneurial, and execution.

#### Connection

One final factor critical for adapting to organizational life is *connection* (the other C in C+MAC model). Indeed, connection or social skills are of growing importance in the labor market [49]. These skills matter because much information, expertise, and resources reside in other people, and because large tasks and goals depend on different people working well together, that is, coordinating but also managing conflict when it arises. The processes and social dynamics that underlie the connection skills are typically studied by social, personality, and organizational psychologists. These include processes such as perspective taking and empathy, social judgment, emotional intelligence, agreeableness, and group dynamics and intragroup/intergroup relations. Research in these disciplines makes several points about the social dimension of life and the importance of the connection skills.

Research on the evolution of intelligence, for example, has proposed that it was in large part the need to navigate social life that pressured for bigger brains and cognitive flexibility [77]. Unlike most other primates, humans also have the tendency to develop relationships beyond pair bonds and kin [78]. Recent research has also shown that being well connected leads to greater personal autonomy [79], increases in motivation [80], improvements in cognitive functioning [81], and better decision making [82]. And good social skills among group members allows groups to perform better [83, 84]. On the other hand, not being able to connect well socially and to get along with others is associated with a host of negative outcomes. These include decreased cognitive functioning [85], wellbeing, and biological health [86]. And in teams and organizations, poor social relationships can easily become amplified and spiral to become more negative [87]. Thus, for different reasons, the skills underlying the connection category are critical for navigating and adapting to one’s social and organizational environments, both as a member of groups and as an individual. The skills making up the connection category in the C+MAC framework include oral & written communication, social tact, empathy, relational, intercultural, and collaboration & teamwork.

Although the skills and the four proposed C+MAC categories are distinct, they function as an interrelated system to help people adapt given their organizational duties and challenges to be solved. For example, understandings gleaned by deploying one’s analytical and problem-solving skills (cognition) can feed motivation skills such as growth orientation and grit. This should be expected because motivation is comprised of both the value an individual attaches to an activity or outcome, but also the assessed feasibility (provided by the cognition skills) that one can perform them [e.g., 88]. The action category includes skills such as initiative and bias for action as well as being entrepreneurial, which partly entails experimenting and trying things out. The action skills are crucial for starting feedback cycles to test whether one’s judgments of a situation are correct. This is particularly important for ensuring that planning and reflection (cognition), for example, are not too comprehensive only to find out later that a plan is not viable [89], as well as to calibrate the optimism (motivation) that can result from planning things into the future [90].

It is also important to point out that the skills for adapting are not necessarily content or process pure. Theoretically, the skills should be related more strongly to one of the C+MAC categories than the others, but some overlap can exist. For example, it is understood that leadership and influence are important skills for getting things done. But they also have interpersonal aspects that should result in some overlap with the connection category. As well, communicating is a skill for interacting with others but one that also has elements of cognition.

Most of the skills tested in this research are those included in the original and more recent NACE reports [91, 92], which grew out of a collaboration between the Conference Board, the Partnership for 21st Century Skills, Corporate Voices for Working Families, and the Society for Human Resource Management [91]. The skills tested here have also been supplemented by the lists reviewed in the comprehensive National Research Council (NRC) report [93], with occasional slight name modifications. There is considerable overlap between the NACE lists and the NRC compilation but taken together they offer a more comprehensive representation of skills across the four categories to be tested in this research. The compilation and distillation resulted in twenty-four skills, six per category (as described above).

The adaptive skills tested in the present research have been called by different names. Examples include 21st century skills or competencies [46, 94–96], lifelong learning competencies [94], key competencies [97], career readiness skills [98], and deeper learning and higher order thinking [93]. Although the collections of *specific* skills differ across frameworks, the focus on broad skills—those skills that apply across different organizational contexts—is the same, but the skill labels and the category labels (and number of categories) vary.

It is important to note that the term “skill,” in its modern usage, may include important personal attributes that matter for work and life success, such as people’s beliefs, values, and attitudes [99]. Examples include growth mindset (belief), professionalism (value and competency), and intercultural sensitivity (attitude and competency). So, it is important to recognize that many skills include these other elements. Further, beliefs and values can be important in the cultivation and application of other skills. For example, even though a person may be a good problem-solver, a poor attitude toward working with others from different social groups or technical backgrounds will likely impede their success at work.

Putting aside the use of word “skill, as well as the diversity in names given to *specific* skills, two challenges confront empirical work on adaptive skills. One is defining the different skills, for example, in terms of how they are manifested in behaviors that can be assessed or reported on by others. The other is diversity in conceptualizations used to group skills [100]. These two challenges are interrelated. Without an internally consistent framework, it is difficult to situate the skills and understand their conceptual boundaries.

But most critical is that of the large and diverse number of theoretical frameworks proposed to organize the varying collections of adaptive skills (see [93] for a review), few, if any, have provided skill definitions and operationalizations. This means that most, if not all, of these frameworks have not been empirically validated, so the challenge in examining these skills scientifically remains. One consequence is that this makes it challenging for companies and other organizations to have a valid model they can use to understand, recognize, and assess the adaptive skills in a systematic way (e.g., in hiring and evaluation) [47, 93, 101].

Thus, the present research provides a comprehensive list of skills for adapting, defines these skills, and proposes a model (the C+MAC framework) for organizing them based on the relevant psychological and organizational literatures. But this work goes further to provide an empirical test of the model, as well as testing an alternative framework. Appendix A provides the list of skills and associated definitions tested in this research.

#### The present research

If people could not discriminate among sensory stimuli, perceptions, ideas, and relate them to superordinate categories, mental life would be mush, a failure to create order out of a chaotic information environment. With no capacity to organize and categorize what one perceives, it would be impossible to remember relationships among events, hypothesize about causal relationships, and form beliefs about the world and oneself. Putting things into categories is the bread and butter of the mind, and it is also a significant part of the engine of science because with no basic ability to categorize, taxonomies cannot be created, nor systems of causal relationships tested. It is also a significant part of organizational life, as organizational members use conceptual frameworks or models as roadmaps to make sense of organizational behavior and organizational events. The purpose of these studies was to test whether people can accurately judge and place the skills for adapting into the categories put forth in the C+MAC framework.

People’s understanding of the different skills was tested in two experiments using a judgment categorization task. Participants were provided with minimal descriptions of the four C+MAC categories (i.e., cognition, motivation, action, connection) and then were asked to judge the extent to which each skill (also defined for them) fit each of the four category descriptions. This follows the procedure used by Craik and Tulving [102] in their classic research examining depth of information processing. If people cannot differentiate among the four categories, then we can conclude that the category distinctions as described are not meaningful to people or that the skill definitions themselves are not valid. But if participants can reliably assign the skills to their designated category (e.g., problem-solving more likely to be assigned to cognition than to motivation, action, and connection categories), then we can conclude that participants have an inherent understanding of the skills as being part of the superordinate category.

## Study 1 methods

### Participants and design

The Health Sciences and Behavioral Sciences Institutional Review Board at the University of Michigan assessed the present research and classified it as exempt. Participants were informed about the study and had to behaviorally consent to participate (pressed “next” button to consent, then continue the task).

The judgment task involved participants judging skills multiple times in response to four C+MAC categories, so the design was within participants. However, for the analyses we compared two dependent means at a time. We recruited 120 participants (paid for their time on Prolific platform; *M*age = 25.88, *SD*age = 8.53; 34.5% female), and 113 completed the study resulting in a 94% response rate. This sample size provides 93% power to detect a small effect (*d =* .30) when comparing two dependent means. The categorization task took on average 14.92 minutes to complete.

Two data checks were included, a consistency check and a cognitive challenge. No participant missed both checks. There were instances of missing data, so the degrees of freedom vary slightly across the presented analyses. The cases with missing data were included to preserve the data structure and power levels. Supplemental analyses, which only examined complete cases, showed similar although slightly weaker results likely due to loss in power.

### Materials and procedure

Participants were told they would be presented with different skills that any person can possess, and that they would be asked questions about the different skills. They were then given a brief description of the four superordinate categories they would use to judge each skill. The categories were *Cognition* (“skills related mainly to mental activity, thinking about information, and mentally figuring things out”); *Motivation* (skills related mainly to having the drive and energy to engage with activities or topics, and a desire to complete goals); *Action* (skills that go beyond motivation and related mainly to taking action to change something or make something happen); and *Connection* (“skills related mainly to social interaction, building relationships, and getting along with others”).

Participants were then presented with the 24 skills and associated definitions, one at a time. The skills themselves were presented in one random order with the constraint that a skill from each of the four categories be presented in each of six blocks. The skills when presented were *not* labeled. The skill of oral / written communication, for example, was given a number (i.e., Skill #4). This was done to prevent participants from using the skill label as a cue to try to infer the superordinate category. In addition, the skill definitions were carefully crafted so that each definition captured specific and essential aspects of that skill. Each skill definition was comprised of three behavioral sub-facets separated by commas, and each definition consisted of 38 words. For instance, for oral / written communication, participants read the following:

“Skill #4: to be able to make presentations and explain topics or events to different audiences, describe one’s perspective or needs so that others know where one is coming from, and write and speak clearly using proper grammar and style.”

After reading through each skill and its definition, participants then judged how well the skill fit the description of each of the four superordinate categories. The placement of the categories below the skills was continuously randomized. Participants made four judgments using 5-point scales that ran from 1 “Not well at all” to 5 “Extremely well.” In total, participants made ninety-six categorization judgments. After completing the task participants were thanked and paid for their time. See Appendix A for the skills and their associated definitions.

## Results and discussion

The data that were analyzed consisted of sixteen means. The means were created by averaging across the six skills (for each category), four different times. For example, the skill of “oral/written communication” (like all the other 23 skills) was judged four times, once in terms of how well it fit the cognition category, the motivation category, the action category, and the connection category. The judgments for “oral/written communication” were aggregated with the judgments for the other five designated connection skills, and this was done for each superordinate category. This resulted in four different means when judging the connection category. These were: the mean of all six “connection” skills when judged for their fit with the *cognition* category; the mean of all six “connection” skills when judged for their fit with the *motivation* category; the mean of all six “connection” skills when judged for their fit with the *action* category; and the mean of all six “connection” skills when judged for their fit with the *connection* category. The same process was carried out for all the other skills. All the means and standard deviations are presented in Table 1, as well as the Cronbach’s alphas.

**Table 1:** Judgments of fit between the different skills with the four different categories in Studies 1 & 2).

	Mean (*sd*)	α
	Study 1 Study 2	Study 1 Study 2
Cognition skills judged for cognition category	4.31 (.72) 4.16 (.62)	.83 .77
Motivation skills judged for cognition category	3.15 (.88) 3.42 (.76)	.82 .78
Action skills judged for cognition category	3.44 (.74) 3.61 (.69)	.73 .74
Connection skills judged for cognition category	3.21 (.79) 3.22 (.80)	.77 .82
Cognition skills judged for motivation category	3.03 (.87) 3.31 (.87)	.82 .83
Motivation skills judged for motivation category	4.17 (.72) 4.06 (.70)	.74 .76
Action skills judged for motivation category	3.58 (.79) 3.68 (.72)	.77 .76
Connection skills judged for motivation category	2.73 (.90) 2.96 (.88)	.84 .85
Cognition skills judged for action category	3.10 (.93) 3.32 (.84)	.82 .80
Motivation skills judged for action category	3.51 (.76) 3.64 (.74)	.72 .77
Action skills judged for action category	3.94 (.63) 3.81 (.65)	.64 .67
Connection skills judged for action category	2.71 (.88) 3.06 (.86)	.83 .82
Cognition skills judged for connection category	2.29 (1.00) 2.67 (.98)	.88 .86
Motivation skills judged for connection category	2.43 (.97) 2.79 (.96)	.83 .86
Action skills judged for connection category	2.98 (.68) 3.22 (.74)	.60 .69
Connection skills judged for connection category	4.45 (.67) 4.31 (.73)	.83 .86

Higher scores indicate higher judged fit with that category.

If participants cannot distinguish reliably among the skills and categories, then the means for the skills (e.g., Cognition skills, Motivation skills, Action skills, and Connection skills) should not differ when judged with respect to any superordinate category (e.g., cognition category or 1^st^ four rows of Table 1). But if the skill definitions and categories are meaningful to participants, they should be able to judge the skills that relate to a category (e.g., Cognition skills judged for the cognition category) higher than they judge the other skills with respect to that same category. This approach keeps the category constant for comparisons. Three comparisons were performed for each category, so Bonferroni corrected *p* < .05 is *p* < .016. All paired sample t-tests were performed bootstrapped to 5000 samples.

I first examined how the skills were judged in terms of the cognition category. The cognition skills were judged as *better fitting the cognition category* than the motivation skills, t(109) = 11.45, p < .001 (*M*diff = 1.14, CI = .94, 1.34, Cohen’s d = 1.05); the action skills, t(104) = 10.09, p < .001 (*M*diff = .86, CI = .69, 1.03, Cohen’s d = .87); and the connection skills, t(110) = 12.52, p < .001 (*M*diff = 1.09, CI = .92, 1.26, Cohen’s d = .92).

The analysis also showed that the motivation skills were judged as *better fitting the motivation category* than the cognition skills, t(112) = 11.21, p < .001 (*M*diff = 1.14, CI = .94, 1.34, Cohen’s d = 1.08); the action skills, t(111) = 7.52, p < .001 (*M*diff = .59, CI = .43, .74, Cohen’s d = .83); and the connection skills, t(109) = 12.90, p < .001 (*M*diff = 1.47, CI = 1.24, 1.69, Cohen’s d = 1.19).

The action skills were judged as *better fitting the action category* than the cognition skills, t(110) = 8.68, p < .001 (*M*diff = .84, CI = .65, 1.03, Cohen’s d = 1.02); the motivation skills, t(110) = 6.01, p < .001 (*M*diff = .42, CI = .28, .56, Cohen’s d = .74); and the connection skills, t(109) = 12.60, p < .001 (*M*diff = 1.23, CI = 1.04, 1.43, Cohen’s d = 1.03).

The final comparisons for the connection category indicated that the connection skills were judged as *better fitting the connection category* than the cognition skills, t(111) = 16.03, p < .001 (*M*diff = 2.15, CI = 1.88, 2.42, Cohen’s d = 1.42); the motivation skills, t(109) = 15.69, p < .001 (*M*diff = 2.03, CI = 1.78, 2.29, Cohen’s d = 1.36); and the action skills, t(108) = 15.77, p < .001 (*M*diff = 1.47, CI = 1.29, 1.66, Cohen’s d = .98).

The results from Study 1 indicate that the skills related to a particular C+MAC category were judged as better fitting that category than the other three categories, thus supporting the proposed four-category framework. The findings indicate that the skills, when defined as done in this research, can be distinguished by people in terms of the four superordinate categories of cognition, motivation, action, and connection.

## Study 2 methods

The pattern of results from Study 1 should not have been obtained if the skills, as defined here, along with the four superordinate categories, are not meaningful to people. However, an important narrative review of adaptive skills published by the National Research Council of the National Academies proposes a three-domain framework, not four [93].

The three domains in the NRC report include the *cognitive* domain, the *intrapersonal* domain, and the *interpersonal* domain [92]. In psychology, “intrapersonal” refers to processes that occur within the person, whereas “interpersonal” refers to processes that occur between and among different people. But the NRC authors also include a third category (cognitive) that is narrower in scope, and one that can be argued is encompassed by the intrapersonal category, as cognition skills refer to processes that occur within the person. So, this creates some confusion. In addition, the breadth of the intrapersonal category invites the inclusion of many disparate skills. This precludes taking advantage of important distinctions among some of the skills if a different categorization scheme was used.

The NRC report authors did not empirically test the 3-domain model but used it to organize the literature they reviewed. Also, they did not formally define the skills. But they did provide descriptions for the three superordinate domains, and these can be used to test their model with the skill definitions used in the present research. Their domain definitions are: “the cognitive domain involves reasoning and memory; the intrapersonal domain involves the capacity to manage one’s behavior and emotions to achieve one’s goals (including learning goals); and the interpersonal domain involves expressing ideas and interpreting and responding to messages from others” (pg. 3).

### Participants and design

The Health Sciences and Behavioral Sciences Institutional Review Board at the University of Michigan classified the present research as exempt. After reading information about the study, participants had to behaviorally consent to participate (pressed “next” button to consent, then continue the task).

140 individuals were recruited for the study, individuals who indicated they managed subordinates (from 1 to < 10). They were paid for their time on the Prolific platform; *M*age = 30.57, *Sd*age = 8.48; 35.8% female). 120 participants completed the study making the response rate 86%. This sample size provides 94% power to detect a small effect (*d =* .30) when comparing two dependent means. The categorization task took on average 20.15 minutes to complete. The study design was within participants, as participants judged the skills seven different times, in response to the three NRC domains [93], and the four C+MAC categories tested in Study 1. For all analyses the design was within participants.

The same data checks used in Study 1 were used in this study. No participant missed both checks. There were some missing data, so degrees of freedom vary slightly across the analyses. As in Study 1, the cases with missing data were included to preserve the data structure and power levels. Supplemental analyses, which only examined complete cases, showed similar yet slightly weaker findings likely due to loss in power.

### Materials and procedure

The materials and procedure were similar to those used in Study 1. Participants were first given a description for each of the three National Research Council’s domains [93]: *cognitive domain* involves reasoning and memory; *intrapersonal* domain involves the capacity to manage one’s behavior and emotions to achieve one’s goals (including learning goals); *interpersonal* domain involves expressing ideas and interpreting and responding to messages from others. And the participants were also given descriptions for the four C+MAC categories used in Study 1 (*Cognition*, *Motivation*, *Action*, and *Connection*), which were always presented after the NRC domain descriptions to ensure no contamination of the C+MAC framework on the three-domain scheme.

As done in Study 1, participants were then presented with the 24 skills and their associated definitions, one at a time using the same random order and block constraints from Study 1. Again, the skills when presented were not labeled to prevent the use of the skill label as a cue to infer the superordinate domain or category.

After reading through each skill and associated definition, participants judged how well the skill fit the description of each of the three NRC domains and then the categories from the C+MAC framework. In this study the domains/categories, when they appeared under a skill on the same page, were not randomized (as they were in Study 1) to provide a conservative test of the C+MAC framework, that is, by having participants always judge the skills in terms of the NRC domains first. So, after reading each skill and its definition, participants made seven judgments using 5-point scales that ran from 1 “Not well at all” to 5 “Extremely well.” In total, participants made 168 categorization judgments.

## Results and discussion

First, this study sought to replicate the Study 1 results with a new sample of participants (see Table 1 for means and standard deviations). Three comparisons were performed for each category, so Bonferroni corrected *p* < .05 is *p* < .016. All paired sample t-tests were performed bootstrapped to 5000 samples.

I first examined the skills in relation to the cognition category. The analyses indicated that the cognition skills were judged as *better fitting the cognition category* than the motivation skills, t(110) = 9.00, p < .001 (*M*diff = .73, CI = .57, .89, Cohen’s d = .86), the action skills, t(113) = 9.56, p < .001 (*M*diff = .55, CI = .44, .67, Cohen’s d = .62), and the connection skills, t(111) = 10.90, p < .001 (*M*diff = .94, CI = .77, 1.11, Cohen’s d = .91).

In examining the motivation category, the analyses revealed that the motivation skills were judged as *better fitting the motivation category* than the cognition skills, t(118) = 8.46, p < .001 (*M*diff = .75, CI = .58, .93, Cohen’s d = .97), the action skills, t(119) = 5.70, p < .001 (*M*diff = .38, CI = .25, .51, Cohen’s d = .73), and the connection skills, t(117) = 10.66, p < .001 (*M*diff = 1.09, CI = .89, 1.30., Cohen’s d = 1.11).

The action category also revealed that the action skills were judged as *better fitting the action category* than the cognition skills, t(118) = 6.97, p < .001 (*M*diff = .49, CI = .35, .63, Cohen’s d = .76), the motivation skills, t(119) = 2.83, p = .005 (*M*diff = .16, CI = .05, .28, Cohen’s d = .63), and the connection skills, t(117) = 8.74, p < .001 (*M*diff = .74, CI = .57, .91, Cohen’s d = .93).

Finally, for the connection category, the analyses indicated that the connection skills were judged as *better fitting the connection category* than the cognition skills, t(113) = 13.22, p < .001 (*M*diff = 1.67, CI = 1.42, 1.92, Cohen’s d = 1.35); the motivation skills, t(116) = 12.68, p < .001 (*M*diff = 1.53, CI = 1.29, 1.77, Cohen’s d = 1.31), and the action skills, t(114) = 11.93, p < .001 (*M*diff = 1.10, CI = .92, 1.28, Cohen’s d = .99). The findings replicate those from Study 1.

### Simultaneous testing of the C+MAC framework with the NRC three-domain conception

To compare the 3-domain approach, two versions of the NRC skill assignments were created to deal with inconsistencies. Out of the C+MAC’s twenty-four skills, only “quantitative” (see Appendix A in S1 Appendix for C+MAC skills) could not be assigned to any version of the NRC skill categorizations. It is also important to remember that the NRC authors did not actually define any of the skills.

Version 1 preserved the original NRC skill assignments, leaving nine skills for their cognitive domain (problem-solving, analytical and detail focus, organizational, planning and reflection, adaptability and flexibility, expertise, oral and written communication, social tact, creative and entrepreneurial), eleven for the intrapersonal domain (adaptability and flexibility, relational, intercultural, growth and mastery orientation, intrinsic engagement, initiative and bias for action, determination and purpose, resilience, execution and production, grit and work ethic, and dedication), and seven skills for the interpersonal domain (oral and written communication, collaboration and teamwork, social tact, empathy, relational, leadership, and influence).

Version 2 removed any skills from Version 1 that overlapped across domains, leaving five skills for the cognitive domain (including problem-solving, analytical and detail focus, organizational, planning and reflection, and expertise), nine skills for the intrapersonal domain (intercultural, growth and mastery orientation, intrinsic engagement, initiative and bias for action, determination and purpose, resilience, execution and production, grit and work ethic, and dedication), and four skills for the interpersonal domain (collaboration and teamwork, empathy, leadership, and influence).

I conducted these analyses to adjudicate between the C+MAC framework and the NRC 3-domain conception of adaptive skills. This was done by first conducting comparisons among the category (domain) consistent skill means. For example, if both conceptions are equivalent, the skills assigned to the *cognition* skills category should be judged as consistent with the description of the *cognition category* (C+MAC framework) to the same degree that the *cognition* skills are judged as consistent with the description of the *cognitive domain* (three-domain scheme), and so forth. But if participants are making distinctions, then the fit of the skills to the category (or domain) should differ. In addition, because the 3-domain scheme has an intrapersonal domain, whereas the C+MAC framework cleaves this domain into a motivation category and an action category, both the category consistent motivation skills (motivation skills judged in relation to motivation category) and action skills (action skills judged in relation to action category) were compared to the domain-consistent intrapersonal skills (intrapersonal skills judged in relation to the intrapersonal category). Two comparisons were performed for each category, so Bonferroni corrected *p* < .05 is *p* < .025. All analyses were performed bootstrapped to 5000 samples.

For the cognition category / cognitive domain comparison, the analysis indicated greater judgments of fit between the skills and the cognition category (C+MAC framework) than the cognitive domain (3-domain scheme: t(112) = 9.14, p < .001 (*M*diff = .43, CI = .34, .53, Cohen’s d = .50). Version 2 of the skills from the 3-domain scheme produced similar results: t(112) = 3.22, p = .002 (*M*diff = .14, CI = .05, .23, Cohen’s d = .48).

In comparing the motivation category skills to the skills assigned to the intrapersonal domain, the analysis yielded a reliable difference, indicating greater judgments of fit between the skills and the motivation category (C+MAC framework) than the intrapersonal domain (version 1 of 3-domain scheme) t(116) = 6.85, p < .001 (*M*diff = .47 CI = .33, .61, Cohen’s d = .74); version 2: (t(116) = 6.19, p < .001 (*M*diff = .44, CI = .30, .58, Cohen’s d = .77).

Comparisons for the action category vs. the intrapersonal domain showed similar results: skills were judged as better fitting the action category (C+MAC framework) than the intrapersonal domain (version 1 of 3-domain scheme) t(116) = 3.64, p < .001 (*M*diff = .22, CI = .10, .35, Cohen’s d = .67); version 2 (t(116) = 3.08, p = .003 (*M*diff = .20, CI = .07, .32, Cohen’s d = .69).

The final comparisons were between the connection category and the interpersonal domain. The skills were judged as better fitting the connection category (C+MAC framework) than the interpersonal domain (version 1 of 3-domain scheme) t(116) = 3.43, p < .001 (*M*diff = .18, CI = .07, .28, Cohen’s d = .55); version 2 (t(116) = 2.71, p = .008 (*M*diff = .15, CI = .04, .27, Cohen’s d = .62).

In summary, the results not only replicated those of Study 1 but additionally indicated that the skills were judged as better fitting the C+MAC framework than the NRC three-domain scheme.

## General discussion

This research intended to provide a better understanding of the skills that help individuals adapt. This involved defining the skills and creating a four-category C+MAC framework in which to situate them. The framework’s categories consist of cognition, motivation, action, and connection, categories supported by considerable research in the psychological and organizational sciences. In addition, the results from the two studies provided empirical support for the C+MAC framework.

The findings from Studies 1 and 2 indicated that participants were more likely to judge skills that fit their theoretically designated category as better exemplars of that category than the other three categories. For example, the cognition skills were judged as better fitting the cognition category than the motivation, action, or connection categories. The same held for the other skills and their designated categories. Subsequent analyses in Study 2 showed that the C+MAC framework provided a better fit to the skills than the influential model put forth by the National Research Council [93].

### Practical implications

Anyone as a member of an organization can develop a general model of how organizations function. These implicit models are applied by individuals to analyze and make sense of organizational events and to problem-solve when a situation does not fit their implicit model. The usefulness of such models depends, though, on how well they square with what is scientifically known about a specific aspect of organizational behavior. But not all areas of organizational behavior have been scientifically plumbed to the same degree, so organizational leaders may be left to rely on idiosyncratic personal experiences and interpretations.

One area of organizational behavior that needs models with firmer scientific footing deals with the skills necessary for individuals to adapt in organizational settings. Many organizations operate with the model that what is needed to perform (not necessarily adapt) to organizational settings is a requisite level of intelligence, signaled by credentials or test scores, and certain personality tendencies [cf. 103]. Skills are usually not considered, maybe due to an assumption that skills are subsumed by intelligence and personality. However, as noted earlier, missing in large part from how intelligence is operationalized are motivation, action, and interpersonal factors [62]. And although some might consider personality as a stand-in for such factors, most personality approaches, even more occupationally specific ones [104], are molar in nature and premised on tendencies far removed from concrete behavior (for a recent exception see [105]). Further, as research has indicated, intelligence and personality, although predictive of organizational outcomes, leave much variance in outcomes unexplained [62, 106, 107]. Thus, there is considerable room for other characteristics such as skills—when placed in a validated model—to be used to understand work outcomes.

The present perspective, based on the C+MAC framework, should thus help inform the models organizations use to understand a large swath of human behavior, as well as perform functions such as recruitment, selection, and evaluation of individuals. The skills are specific and defined in terms of how they are manifested through behavior, thought, and feelings; the skills can be understood in terms of a validated conceptual framework that it easy to communicate; and the skills represent spheres of individual performance that companies indicate represent capacities they seek in organizational members [60, 98].

Independent measures (not necessarily performance based) exist for some of the adaptive skills from the C+MAC framework, such as bias for action [108], leadership [109], perspective taking [110], and grit [111], for example. But in organizational settings, assessing each skill in the C+MAC framework, even with abbreviated measures, might prove challenging given the number of skills to be assessed (also see [101]). One approach that seems more practicable is to assess the adaptive skills using a 360 methodology. With skills defined concretely in terms of observable behavior, individuals can report on the degree to which they manifest behaviors associated with each of the different skills. These same definitions can then be used by evaluators to judge the person in question based on their own experiences and observations. So instead of a numerical test score, individuals can gain easy-to-use feedback based on the degree to which they demonstrate the different skills. In addition, they get the opportunity to ascertain how well their self-judgments are calibrated with how other organizational members see them. Given that many individuals tend to see themselves as more skilled than they are [112], getting this type of social feedback is powerful for increasing self-awareness [113].

### Theoretical implications

Adaptive skills are central to the interface between individual employees and the tasks they perform. But the skills also matter for the other elements of the organizational transformation process, and changes that occur in these elements. Although it is possible to focus on each C+MAC skill and relate it to each of the interfaces of the organization transformation process, this is beyond the scope of this discussion and would require significant space to be done properly. But matters can be simplified by asking: Does being part of the formal organization with assigned roles and duties rely on adaptive skills? Or, do adaptive skills play a role in the informal organization with its organically formed relationships and influence patterns? In both cases, the answer is yes. For example, all organizational members need cognition skills to understand the formal aspects of their organization and their duties, but also to understand how the informal organization can help or hinder what the formal organization is trying to get done. Further, given that organizational performance is influenced by the congruence or fit between the interfaces [28], organizational members can use their cognition skills to diagnose problems or suboptimal processes to help amplify what the formal organization is trying to accomplish. This could also involve using action skills (e.g., creative & entrepreneurial, influence) to come up with ideas and to get other organizational members to try new ways of doing the work, as well as connection skills (e.g., empathy, social tact) to work well with others and to understand why some organizational members may be hesitant to change how they do their work.

The skills from the C+MAC framework also matter for members at other organizational levels (managers, members of executive team). A good leader must understand the environment in which their organization operates—the threats, constraints, demands, and opportunities. The cognition skills serve as a platform for applying tools for analyzing the different aspects of the environment, for planning, and for problem-solving. Some of the motivation skills such as growth mindset underpin approaching opportunities for learning (as an individual leader, or the organization as a whole), which should help keep status quo thinking at bay to better assess, for example, the organization’s opportunities and the competitive landscape. Determination and purpose also ensure one is developing goals and vision to further the effectiveness and growth of the organization, and the actions skills such as leadership, influence, and execution ensure that goals are pursued with buy-in from others and with concrete objectives to make them real. And the connection skills such as tact and communication help leaders network effectively and represent the organization to external parties and shareholders, as well as keep organizational members informed about strategy and progress toward goals. Thus, not only are adaptive skills relevant across different organizational spheres, they also represent a resource that facilitates performance for all organizational members.

### Surviving versus thriving

The concept of opportunity has received much attention as well as spirited discussion at the level of organizations and creation of new organizational forms [e.g., 114, 115]. But little work has been done on how individuals think of, construct, and pursue opportunities for themselves. Consequently, there is little theoretical development and research on how skills factor into the creation of opportunities. Earlier I discussed how skills, along with representing competencies, also include attributes such as values, attitudes, and beliefs [99]. In the introduction I provided the example of a person with good problem-solving skills but a poor attitude toward working with others from different technical backgrounds. If they held a different attitude toward working with others, or toward individuals from different backgrounds, this could help amplify their learning opportunities as well as feed their collective efficacy. But to get even closer to a conception of individual opportunity, though, we also need to consider market realities in addition to people’s skills and desires. The likelihood that an individual can pursue their desires by manifesting their skills may be low because there is a limited number of occasions for doing so. Let’s return to the example of the good problem-solver. Given that a greater share of work in organizations today is done in teams [116], this makes it less likely this individual will be rewarded solely based on their work as the market realities for providing value on one’s own are diminishing.

In summary, skills matter for adapting to organizational environments. But in addition to surviving, skills should work in conjunction with other skills or skill attributes (values, attitudes), as well as with market realities, to give shape to the opportunity landscape a person is able to experience in their work. Thus, awareness of how different skills and their attributes interact, along with some strategic thinking about what one has to offer and likelihood of it being valued, can transform one’s adaptation from mere survival to thriving and growth.

### Limitations

The present work acknowledges the importance of but does not include skills related to information and communication technology (ICT). ICT skills are narrower in scope [101], but it is nevertheless understood that organizations will likely continue to assume (if not explicitly require) some capacity for deploying information and communication technology related skills. In addition, it could be that because of IC-related technologies, to adapt, people will need to develop other skills not currently included in the C+MAC framework. One possibility is dealing with distractions, and relatedly, with the spillover that occurs between work and life. For now, let’s call this skill “distraction control.” The skill would likely be characterized as a cognition skill as it draws on cognitive processes such as attention control and working memory [e.g., 117], which have been proposed as critical aspects of leader performance in organizations [53]. IC technologies matter for distraction in different ways. They matter because of the introduction of platforms to facilitate remote work, and the capacity of email to interrupt focus and concentration on actual work in the service of workflow management [118]. Adaptation generally depends on analyzing organizational situations and problem-solving when challenges arise, as well as pursuing goals and meeting expectations. The inability to control distractions in a work world that is overloaded with information will likely impair for some individuals the ability to deal with these organizational demands. In terms of the broader C+MAC framework, though, the hope is that the framework is conceptualized in such a way that it can accommodate skills relevant to the four categories but at the same time is not so general that all new skills can be placed within it.

The theoretical and methodological focus of the present research uses a “top-down” categorization of the different skills. Individuals are not asked to judge themselves or to have others judge them in terms of the skills. Instead, borrowing a paradigm from cognitive psychology, individuals explicitly judge how well a skill fits a defined category and do so multiple times. Such categorization occurs regularly in organizational contexts, for example, as organizations select, develop, and promote employees based on the skills they are thought to have, and as organizational members communicate who has certain skills and who needs to cultivate them. In future research it would nevertheless be useful to further validate the C+MAC framework by having a large sample of participants rate themselves (or have others rate them) on the different skills, and then apply statistical procedures such as factor analysis to test the four-category pattern in judgments of self or others.

Finally, it is important to acknowledge the potential limitations of relying on on-line participants. On-line participants could differ in various ways from individuals who could be recruited via other methods, or who are assessed and observed in richer contexts provided by work environments. In addition, bringing in more context should allow for asking other relevant questions, such as how the present framework predicts adaptive work outcomes and performance above and beyond alternative models.

## Conclusion

Broad skills, as organized in the C+MAC framework, matter for individuals’ ability to adapt to organizational environments, both in terms of meeting the demands of their portfolio of projects (surviving) but also thriving, that is, learning and growing from responding to change. Because organizations get their work done in large part through people, organizational performance and adaptation also rely on individuals’ skills, as skills help bridge individuals to their tasks, but also tasks and individuals to the other elements of the organizational transformation process. Not all possible skills are covered in the framework, and future shocks to society, the economy, and to how people work will likely introduce the need for others. Nevertheless, the empirical support for these adaptive skills and the C+MAC framework should give members of organizations and leaders a useful and validated model for making sense of a critical aspect of how organizations get their work done.

## Supporting information

S1 File (PDF)

S1 Appendix (PDF)
