# Supplementary material for: The skills that help employees adapt: Empirical validation of a four-category framework
Source: PLoS One. 2023 Feb 24;18(2):e0282074. doi: 10.1371/journal.pone.0282074 (PMC9955657; doi:10.1371/journal.pone.0282074)
Supplement: S1 File — (PDF) [file pone.0282074.s001.pdf]

Study 1 Results Without Missing Data

The cognition skills were judged as *better fitting the cognition category* than the motivation skills, $t(96)=10.79$, $p < .001$ (Mdiff = 1.15, CI = .94, 1.37, Cohen's d =1.05); the action skills, $t(96)=9.43$, $p < .001$ (Mdiff = .84, CI = .67, 1.02, Cohen's d =.88); and the connection skills, $t(96)=11.70$, $p < .001$ (Mdiff = 1.08, CI = .90, 1.26, Cohen's d =.91).

The analysis also showed that the motivation skills were judged as *better fitting the motivation category* than the cognition skills, $t(96)=10.56$, $p < .001$ (Mdiff = 1.15, CI = .93, 1.37, Cohen's d =1.07); the action skills, $t(96)=7.04$, $p < .001$ (Mdiff = .58, CI = .41, .74, Cohen's d =.81); and the connection skills, $t(96)=12.01$, $p < .001$ (Mdiff = 1.47, CI = 1.23, 1.72, Cohen's d =1.21).

The action skills were judged as *better fitting the action category* than the cognition skills, $t(96)=8.02$, $p < .001$ (Mdiff = .82, CI =.62, 1.02, Cohen's d =1.01); the motivation skills, $t(96)=5.99$, $p < .001$ (Mdiff = .43, CI =.29, .57, Cohen's d =.71); and the connection skills, $t(96)=12.08$, $p < .001$ (Mdiff = 1.22, CI =1.02, 1.43, Cohen's d =1.00).

The final comparisons for the connection category indicated that the connection skills were judged as *better fitting the connection category* than the cognition skills, $t(96)=15.15$, $p < .001$ (Mdiff = 2.16, CI =1.88, 2.45, Cohen's d =1.41); the motivation skills, $t(96)=14.68$, $p < .001$ (Mdiff = 2.01, CI =1.74, 2.29, Cohen's d =1.35); and the action skills, $t(96)=15.19$, $p < .001$ (Mdiff = 1.46, CI =1.27, 1.65, Cohen's d =.94).

The results from Study 1 indicate that the skills related to a particular C+MAC category were judged as better fitting that category than the other three categories, thus supporting the proposed four-category framework.

Study 2 Results Without Missing Data

The analyses indicated that the cognition skills were judged as *better fitting the cognition category* than the motivation skills, t(90)=8.02, p < .001 (*M*diff = .74, CI = .56, .92, Cohen's d = .88), the action skills, t(90)=8.01, p < .001 (*M*diff = .54, CI = .41, .67, Cohen's d = .64), and the connection skills, t(90)=9.76, p < .001 (*M*diff = .96, CI = .77, 1.16, Cohen's d = .94).

In examining the motivation category, the analyses revealed that the motivation skills were judged as *better fitting the motivation category* than the cognition skills, t(90)=7.41, p < .001 (*M*diff = .73, CI = .53, .93, Cohen's d = .94), the action skills, t(90)=4.87, p < .001 (*M*diff = .36, CI = .21, .51, Cohen's d = .71), and the connection skills, t(90)=9.98, p < .001 (*M*diff = 1.11, CI = .89, 1.34., Cohen's d = 1.06).

The action category revealed that the action skills were judged as *better fitting the action category* than the cognition skills, t(90)=5.69, p < .001 (*M*diff = .48, CI = .31, .64, Cohen's d = .80), marginally so for the motivation skills, t(90)=1.50, p = .07 (*M*diff = .10, CI = -.03, .23, Cohen's d = .63), and the connection skills, t(90)=7.78, p < .001 (*M*diff = .78, CI = .58, .98, Cohen's d = .96).

Finally, for the connection category, the analyses indicated that the connection skills were judged as *better fitting the connection category* than the cognition skills, t(90)=11.87, p < .001 (*M*diff = 1.74, CI = 1.45, 2.04, Cohen's d = 1.40); the motivation skills, t(90)=11.66, p < .001 (*M*diff = 1.61, CI = 1.33, 1.88, Cohen's d = 1.31), and the action skills, t(90)=10.83, p < .001 (*M*diff = 1.16, CI = .95, 1.38, Cohen's d = 1.03). The findings thus replicate those from Study 1.

Simultaneous Testing of the C+MAC framework with the NRC Three-Domain Conception

For the cognition category / cognitive domain comparison, the analysis indicated greater judgments of fit between the skills and the cognition category (C+MAC framework) than the cognitive domain (3-domain scheme: $t(90)=7.91$, $p < .001$ (*M*diff = .43, CI = .32, .54, Cohen's d = .52). Version 2 of the skills from the 3-domain scheme produced similar results: $t(90)=2.75$, $p = .01$ (*M*diff = .13, CI = .03, .23, Cohen's d = .46).

In comparing the motivation category skills to the skills assigned to the intrapersonal domain, the analysis yielded a reliable difference, indicating greater judgments of fit between the skills and the motivation category (C+MAC framework) than the intrapersonal domain (version 1 of 3-domain scheme) $t(90)=5.90$, $p < .001$ (*M*diff = .48 CI = .33, .65, Cohen's d = .78); version 2: ($t(90) = 5.25$, $p < .001$ (*M*diff = .45, CI = .29, .63, Cohen's d = .81).

Comparisons for the action category vs. intrapersonal domain showed similar results: skills were judged as better fitting the action category (C+MAC framework) than the intrapersonal domain (version 1 of 3-domain scheme) $t(90)= 3.04$, $p < .003$ (*M*diff = .23, CI = .09, .38, Cohen's d = .72); version 2 ($t(90) = 2.53$, $p = .007$ (*M*diff = .20, CI = .05, .35, Cohen's d = .74).

The final comparisons were between the connection category and the interpersonal domain. The skills were judged as better fitting the connection category (C+MAC framework) than the interpersonal domain (version 1 of 3-domain scheme) $t(90)= 2.80$, $p = .007$ (*M*diff = .16, CI = .05, .27, Cohen's d = .55); version 2 ($t(90) = 2.26$, $p = .025$ (*M*diff = .15, CI = .02, .28, Cohen's d = .63).

Table 1: Judgments of fit between the different skills with the four different categories in Studies 1 & 2). Higher scores indicate higher judged fit with that category.

	Mean (*sd*)		α	
	Study 1	Study 2	Study 1	Study 2
Cognition skills judged for cognition category	4.32 (.74)	4.16 (.64)	.85	.80
Motivation skills judged for cognition category	3.16 (.86)	3.42 (.77)	.81	.78
Action skills judged for cognition category	3.47 (.74)	3.62 (.69)	.74	.74
Connection skills judged for cognition category	3.24 (.76)	3.20 (.81)	.75	.83
Cognition skills judged for motivation category	3.06 (.84)	3.32 (.89)	.80	.83
Motivation skills judged for motivation category	4.21 (.72)	4.05 (.70)	.74	.75
Action skills judged for motivation category	3.63 (.77)	3.68 (.74)	.77	.77
Connection skills judged for motivation category	2.73 (.90)	2.93 (.87)	.84	.84
Cognition skills judged for action category	3.12 (.93)	3.32 (.89)	.82	.82
Motivation skills judged for action category	3.51 (.75)	3.70 (.74)	.72	.77
Action skills judged for action category	3.94 (.62)	3.79 (.66)	.63	.66
Connection skills judged for action category	2.72 (.87)	3.01 (.88)	.82	.83
Cognition skills judged for connection category	2.30 (.99)	2.58 (1.01)	.88	.87
Motivation skills judged for connection category	2.45 (.98)	2.72 (.98)	.84	.86

Action skills judged for connection category	3.01 (.66)	3.16 (.76)	.58	.71
Connection skills judged for connection category	4.47 (.65)	4.33 (.73)	.82	.87
