# Supplementary material for: The skills that help employees adapt: Empirical validation of a four-category framework
Source: PLoS One. 2023 Feb 24;18(2):e0282074. doi: 10.1371/journal.pone.0282074 (PMC9955657; doi:10.1371/journal.pone.0282074)
Supplement: S1 Appendix — (PDF) [file pone.0282074.s002.pdf]

Appendix A

Cognition

Problem-Solving, to be able to consider diverse sources of knowledge when reasoning about novel events, generate and link together different ideas to create possible solutions, and iterate through different tactics until finding one that fits the challenge at hand.

Planning and Reflection, to be able to think about the future and determine what activities to pursue, assess one's current progress on tasks and projects, and outline the steps, time, and resources that will be necessary for achieving those future outcomes.

Expertise, to be able to access and command significant amounts of knowledge in one's field of study, recognize when that knowledge is relevant for understanding events, and utilize that understanding and previously learned procedures for approaching tasks and projects.

Analytical and Detail Focus, to be able to break down projects into smaller parts, comprehend the different pieces of a process or procedure to diagnose problems, and identify elements that are left out from the way a task or project is defined.

Quantitative, to be able to understand data and translate how numbers can represent observations of events in life, determine relevant mathematical procedures or statistical analyses to apply to data, and interpret findings to draw insights and make valid recommendations.

Organizational ability, to be able to classify and arrange tasks that are to get done, decide the order in which assignments are to be performed, and judge the amount of time necessary so projects are completed in a timely fashion.

Motivation

Intrinsic Engagement, to be able to find aspects to one's work that are meaningful, stick with a topic or project for extended periods without getting distracted, and enjoy taking on challenging activities even when not directly applicable to one's ambitions.

Grit and Work Ethic, to be able to generate the effort needed to fulfill one's responsibilities, pursue goals that extend over long periods of time and not give in to distractions, and persist with priorities even when recognition is not immediately forthcoming.

Resilience, to be able to manage frustrations and annoyances that arise in one's work or life, bounce back quickly when effort on projects comes across hurdles, and remain unflustered when the duties one is accountable for become more taxing.

Determination and Purpose, to be able develop enduring intentions that fuel and give direction to one's work, pursue aims in life that are of personal value, and remain steadfast in the face of setbacks or when progress on projects is slow.

Dedication, to be able to put forth effort to comply with and get satisfaction from assignments that are not necessarily of one's choosing, develop ownership of tasks and projects and identify with them, and show commitment to one's work.

Growth and Mastery Orientation, to be able to approach tasks with the intention of performing them well versus just getting them done, seek assignments requiring more experience than one currently has, and undertake challenging topics even when it can result in mistakes.

Action

Leadership, to be able to clarify what's to be done and assign duties and tasks accordingly, instruct others so they become more competent and confident, and initiate efforts to change how things are done when old ways are ineffective.

Influence, to be able to get others to support one's proposals, induce peers or co-workers to take on new activities or change the way they are currently doing things, and provide feedback to ensure agreed upon tasks are completed.

Behavioral Flexibility, to be able to attempt tasks and activities that are new and one has little experience with, work on assignments that have no formula or recipe for their solution, and adjust how to complete projects when conditions change.

Initiative and Bias for Action, to be able to jump in to get a project off the ground, start work on projects without needing to understand all the details, and engage in trial and error to test different ways of resolving unanticipated glitches.

Creative and Entrepreneurial, to be able to challenge the status quo and pursue opportunities to make things better, experiment with ways of doing things using different physical or intellectual materials, and take necessary risks to determine the appeal of one's inventions.

Execution, to be able to implement activities and processes and take a plan from concept to reality, perform procedures to complete tasks and move projects forward, and check progress on one's activities to ensure work is finished on time.

Connection

Oral/Written Communication, to be able to make presentations and explain topics or events to different audiences, describe one's perspective or needs so that others know where one is coming from, and write and speak clearly using proper grammar and style.

Empathy, to be able to put oneself in someone else's situation to get a sense of their experiences, use one's own perspective to anticipate what others believe or intend to do, and be sensitive to others' feelings and emotions.

Intercultural, to be able to interact with people from different social groups, be interested in people from other parts of the country or the world, and consider how people's backgrounds can be a factor in their decisions and behavior.

Collaboration and Team Work, to be able to appreciate the different skills others bring to a project, have candid discussions about the quality of the team's work or to resolve conflicts, and pursue common goals even when colleagues have different work styles.

Relational, to be able to treat others well regardless of who they are, make choices that consider and anticipate the effect of one's decisions on others, and behave in ways that gives others confidence one can be depended on.

Social Tact, to be able to listen closely and respectfully when others are speaking, behave in ways that fit with and are appropriate to different social situations, and keep calm, cool, and collected even when conversations become touchy or irritating.
